# Antitumor Activity of Doxorubicin-Loaded Carbon Nanotubes Incorporated Poly(Lactic-Co-Glycolic Acid) Electrospun Composite Nanofibers

**DOI:** 10.1186/s11671-015-1044-7

**Published:** 2015-08-26

**Authors:** Yuan Yu, Lijun Kong, Lan Li, Naie Li, Peng Yan

**Affiliations:** Department of Biochemistry and Molecular Biology, Binzhou Medical University, Yantai, Shandong 264003 People’s Republic of China; Medicine and Pharmacy Research Center, Binzhou Medical University, Yantai, Shandong People’s Republic China; Department of Physics, Binzhou Medical University, Yantai, Shandong 264003 People’s Republic China

**Keywords:** Electrospun nanofiber, Poly(lactic-co-glycolic acid), Carbon nanotubes, Drug release, Antitumor efficacy

## Abstract

The drug-loaded composite electrospun nanofiber has attracted more attention in biomedical field, especially in cancer therapy. In this study, a composite nanofiber was fabricated by electrospinning for cancer treatment. Firstly, the carbon nanotubes (CNTs) were selected as carriers to load the anticancer drug—doxorubicin (DOX) hydrochloride. Secondly, the DOX-loaded CNTs (DOX@CNTs) were incorporated into the poly(lactic-co-glycolic acid) (PLGA) nanofibers via electrospinning. Finally, a new drug-loaded nanofibrous scaffold (PLGA/DOX@CNTs) was formed. The properties of the prepared composite nanofibrous mats were characterized by various techniques. The release profiles of the different DOX-loaded nanofibers were measured, and the in vitro antitumor efficacy against HeLa cells was also evaluated. The results showed that DOX-loaded CNTs can be readily incorporated into the nanofibers with relatively uniform distribution within the nanofibers. More importantly, the drug from the composite nanofibers can be released in a sustained and prolonged manner, and thereby, a significant antitumor efficacy in vitro is obtained. Thus, the prepared composite nanofibrous mats are a promising alternative for cancer treatment.

## Background

Up to date, cancer is still one of the deadliest killers to human lives, because both the incidence and mortality rates of cancers (including lung, stomach, liver, prostate, colorectum, breast cancers, and so on) are continuously rising [1, 2]. Unfortunately, it can be said that, as of yet, despite tremendous efforts have been made in the cancer therapy, there is still no efficient methods for prevention of cancer relapse, as well as the prevention of their spread and metastasis [3]. A wholesale and thorough cure for cancers remains elusive for a number of reasons. Some of these reasons including late stage diagnosis, inadequate resection during surgery, and cancer cell migration often lead to cancer recurrence [4, 5]. Local cancer recurrence after “curative” treatment remains a major clinical problem for most cancers. As we all know, current therapeutic approaches for malignant tumors include surgery, radiotherapy, chemotherapy, hyperthermia, immunotherapy, hormone therapy, stem cell therapy, and combinations of these modalities [6]. Chemotherapy, as a general therapeutic approach, has been widely investigated to treat a variety of malignant cancer cells [7, 8]. Doxorubicin (DOX), a class I anthracycline antibiotic, is an excellent broad-spectrum anticancer drug for treating many types of cancers [9]. It can effectively kill cancerous cells by damaging DNA and its replication via the mechanisms of intercalation between nucleotides, inhibition of topoisomerase II, and generating oxygen free radicals [10]. However, the clinical application of free DOX is known to have short life and low therapeutic index, and a large dosage of administration dosages are required to achieve the desirable therapeutic effect, which also causes severe toxicity to normal tissues, for example, cardiotoxicity and myelosuppression [10, 11]. Therefore, the development of a suitable carrier system for resisting local cancer recurrence is necessary.

Nanotechnology as an emerging technology may be a reasonable choice and an effective strategy for solving the above problem. According to the report from the National Cancer Institute (NCI) in USA, nanotechnology seeks to exploit distinct technological advances towards cancer prevention and treatment [12]. Electrospinning is a versatile technique to fabricate two-dimensional nanofibers. Electrospun nanofibers have recently received significant attentions in biomedical applications because they possess large surface area-to-volume ratio, high interfiber porosity with tunable pore size, low hindrance for mass transfer, flexible handling, adjustable morphology, and well mechanical strength, which would make nanofibers use as therapeutic patch for drug delivery [13, 14]. The controlled drug release from drug-loaded nanofibrous mat at a rate according to the need could be realized by properly designed architecture, porosity, fiber diameter, drug incorporation manner, and composition of nanofibers [15]. In addition, the medicated nanofibrous products can be easily set to the targeted site by adjusting their morphology [15]. Thus, the drug-loaded nanofibers can be targeted delivery to the desired target tissues with controlled release where drug is released in a certain way for preventing local tumor recurrence after surgery. Aliphatic biodegradable polyester, such as poly(lactic acid-co-glycolic acid) (PLGA), is widely used to form electrospun nanofibers in various clinic and xsimplebiomedical research applications because it has excellent biocompatibility and biodegradability [10, 16]. However, the simple introduction of drug into polymer matrix always leads to inevitable burst drug release, since the drug molecules might migrate on or near the fiber surfaces because of the high ionic strength in drug/polymer solution and the rapid evaporation of the solvent during electrospinning. To address this limitation, it is highly desirable to develop efficient nanocarrier-mediated nanofibrous delivery systems which may serve as barrier for improving the safety of anticancer drugs and avoid drug premature burst release under physiological conditions [7, 17–21]. In fact, several nanoscale carriers such as mesoporous silica nanoparticles [22], hydroxyapatite [10], and liposome [23] have recently been incorporated into electrospun nanofibers for potential anticancer therapy, from which the prolonged drug release with tunable drug release kinetics could be achieved. Carbon nanotubes (CNTs), which are rolled-up seamless cylinder of graphene sheets, have gained extensive attention in the past decade [24]. CNTs are classified as single-walled carbon nanotubes (SWNTs) or multi-walled carbon nanotubes (MWNTs), which depend on the number of graphene layer which a single nanotubes is composed [24]. In recent years, efforts have also been devoted to explore the potential biomedical application of CNTs. Benefitting from their superior heir distinct properties, especially, the biocompatibility in the physiological condition, CNTs have been one of the most promising inorganic nano-sized vectors for anticancer drugs, proteins, genetic therapeutics, and biological imaging agent delivery [8, 24, 25]. Up to now, the development of CNTs-doped PLGA nanofibers for cancer therapy has not been reported to the best of our knowledge. The objective of this work, therefore, was to examine the hypothesis of preparing a sustained anticancer drug release system by doping drug-loaded CNTs into PLGA nanofibers.

In this present work, we have successfully fabricated DOX-loaded PLGA/CNTs composite nanofibrous mats by using electrospinning technique and then have assessed its antitumor efficacy against HeLa cells. The morphology and structure of electrospun nanofibers were characterized. After the evaluation of the drug loading capacity and efficiency, the in vitro release characteristics of DOX from the composite nanofibers were also measured. And further cytotoxicity experiments demonstrate that the drug-loaded nanofibers exhibit obviously therapeutic effect for cancer cell, implying that it may become a novel therapy strategy for preventing local cancer recurrence in future cancer therapy.

## Methods

### Materials

PLGA with lactic acid/glycolic acid ratio of 75:25 (Mw = 110 kDa) was purchased from Daigang Biomaterials Inc. (Jinan, China). Carbon nanotube (CNTs) was purchased from Chengdu Organic Chemicals Co., Ltd. Doxorubicin (DOX) hydrochloride was purchased from Beijing Huafeng United Technology Co., Ltd. HeLa cells were supplied by Institute of Biochemistry and Cell Biology (the Chinese Academy of Sciences, Shanghai, China). Dulbecco’s Modified Eagle’s Medium (DMEM), fetal bovine serum (FBS), 3-(4,5-dimethylthiazol-2-yl)-2,5-diphenyltetrazoliumbromide (MTT), trypsin, penicillin (100 U/mL), and streptomycin (100 mg/mL) were all purchased from Shanghai Yuanxiang Medical Equipment Co., Ltd. All other chemicals were obtained from Sino-pharm Chemical Reagents Co., Ltd. (Shanghai, China).

### Fabrication of DOX-loaded CNTs

The DOX-loaded CNTs (DOX@CNTs) were fabricated by the previously described method with some modifications [10]. Briefly, DOX aqueous solution (1 mg/mL) was prepared, and 20 mg CNTs was dispersed into DOX solution. The mixture was stirred under dark conditions for 12 h and vacuumed slowly at room temperature for 3 h. The DOX@CNTs were collected by centrifugation (10,000 rpm, 10 min) and washed with phosphate-buffered saline (PBS) (pH = 7.4) solution to remove the dissociative DOX. The obtained DOX@CNTs was vacuum-dried at room temperature and stored by sealing for future use. To evaluate the loading efficiency of DOX, the supernatant was collected, and the DOX concentration in the supernatant was analyzed by using UV-vis spectrophotometer at 488 nm. The loading percentage of DOX in CNTs was calculated as follows:

Loading percentage (%) = (initial weight of DOX−residual weight of DOX)/weight of DOX-loaded CNTs × 100 %.

### Preparation of PLGA/DOX@CNTs Nanofibers

The PLGA/DOX@CNTs composite nanofibers were fabricated by using a blend electrospinning. Firstly, the prepared DOX@CNTs was completely dispersed in hexauoroisopropanol (HFIP) and the PLGA was added into the above solution at 20 *w*/*v* %, and then stirred thoroughly to form a homogenous spinning solution. The solution was placed into a 5-mL plastic syringe with an 18-gauge blunt-ended needle. The electrospinning parameters were set as follows: the applied voltage of 20 kV, the collection distance of 15 cm, and the solution flow rate of 1.0 mL/h controlled by a syringe pump (789100C, Cole-Parmer Instruments, USA). The collected nanofibers were vacuum-dried at least 72 h to remove the residual solvent before further use.

The CNTs content in the nanofibers were 0.5, 1, and 2 wt. % (CNTs relative to PLGA), and the corresponding DOX contents in nanofibers can be calculated from the loading efficiency of CNTs. For comparison, the PLGA/DOX composite nanofibers were also prepared by electrospinning with the same DOX contents.

### Characterization

The morphology of the composite nanofibers was observed by a scanning electron microscope (SEM, Hitachi TM-1000, Japan), and the distribution of DOX@CNTs in the nanofibers was characterized by a transmission electron microscope (TEM, JEM-2100, Japan) at an operating voltage of 200 kV. At least 40 nanofibers were selected from different SEM image, and the nanofiber diameter was measured using Image J 1.40 G software (NIH, USA). The fluorescent images of the different composite nanofibers were observed by a fluorescence microscope (Nikon TS100, Japan). The thermogravimetric analysis (TGA) was conducted on a thermal analyzer (TG 209 F1, Germany) from the room temperature to 600 °C at a heating rate of 10 °C/min. The tensile testing of the composite nanofibers with a planar area of 50 × 10 mm was measured using a universal material tester (H5K-S, Hounsfield, UK) with a cross-head speed of 10 mm/min.

### In Vitro Drug Release

The DOX release behaviors from the DOX-loaded samples including PLGA/1.5 % DOX and PLGA/1.5 % DOX@2 % CNTs electrospun nanofibers were carried out in PBS at pH 7.4. Briefly, a certain amount of the different DOX-loaded samples with the same content of DOX were put in a dialysis bag (cutoff molecular weight 7000 Da), and the bag was immersed in 15 mL of PBS (pH 7.4) at 37 °C in a thermostatted shaker with shaking at a rate of 100 rpm. At selected time intervals, the release medium (5 mL) was taken out and supplied with the same volume of fresh PBS. The concentration of DOX in the release media was determined by recording the absorbance of DOX at 480 nm using UV-vis spectrophotometer.

### Cytotoxicity Evaluation of Different DOX-Loaded Samples

HeLa cells were cultured in the DMEM medium supplemented with 10 % FBS 100 U/mL penicillin and 100 μg/mL streptomycin. The cells were cultured at 37 °C in a humidified incubator containing 5 % CO_2_. MTT assay was employed to evaluate the viability of the HeLa cells cultured with different samples. For all experiments, cells were harvested by using trypsin solution and resuspended in the fresh DMEM medium. Before cell seeding, the samples were sterilized under UV light for 3 h and washed with PBS for three times.

For MTT assay, HeLa cells at a density of 1 × 10^4^ cells/well were seeded in 24-well plates and cultured for 12 h to allow cells to attach. Then, the medium was replaced with a fresh medium (negative control) and the medium containing the PLGA/2 % CNTs, free DOX (positive control), PLGA/1.5 % DOX, and PLGA/1.5 % DOX@2 % CNTs composite nanofibers, and the total DOX concentrations was designed 10 mg/mL, 25 mg/mL, and 50 mg/mL. After the cells were cultured for 24 h, the cells were washed twice with PBS and the 360-μL fresh culture medium and 40-μL MTT solution (5 mg/mL in PBS) were then added to each well. The cells were incubated for another 4 h. Thereafter, the 400 μL suspension was discarded, and 400 μL of DMSO was added to each well to completely dissolve the precipitate by stirring for 15 min. Then, 100 μL of the supernatant was transferred to 96-well microplates, and the OD value was measured with a microplate reader (MK3, Thermo, USA) at 492 nm. The relative cell viability was calculated by dividing the mean OD value of the control group, and the average value was obtained from five parallel samples.

For confocal microscopy observation, HeLa cells were seeded into 24-well plates at a density of 1 × 10^4^ cells/well and incubated for 24 h. Then, the culture medium was removed, and the cells were incubated with PLGA/2 % CNTs, free DOX, PLGA/1.5 % DOX, and PLGA/1.5 % DOX@2 % CNT composite nanofibers (DOX concentrations at 25 μg/mL) for another 24 h. After washing with PBS twice, the cells were fixed with 4 % paraformaldehyde for 10 min. The cells were then permeabilized in 0.1 % Triton X-100 in PBS for 5 min, followed by blocking with 1 % BSA for 20 min. The F-actin of cells was stained by using Alexa Fluor 488® phalloidin solution for 10 min. Finally, all samples were washed several times with PBS and observed by confocal laser scanning microscope (CLSM).

## Results and Discussion

### Characterization of Composite Nanofibers

**Fig. 1:**
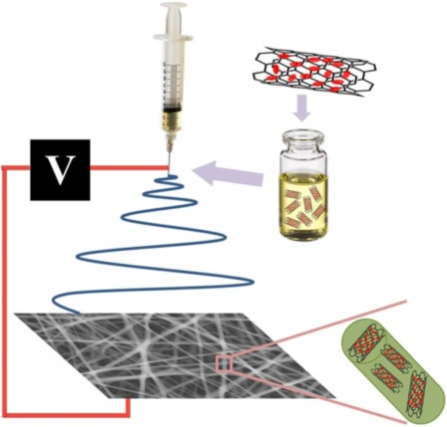
Schematic illustration for the process of fabrication of PLGA/DOX@CNTs electrospun composite nanofibers

Figure 1 shows the brief fabrication process of PLGA/DOX@CNTs composite nanofibers. DOX was firstly loaded into CNTs by the physical absorption. Subsequently, the obtained DOX@CNTs was mixed with the PLGA solution to fabricate PLGA/DOX@CNTs composite nanofibers by electrospinning.

**Fig. 2:**
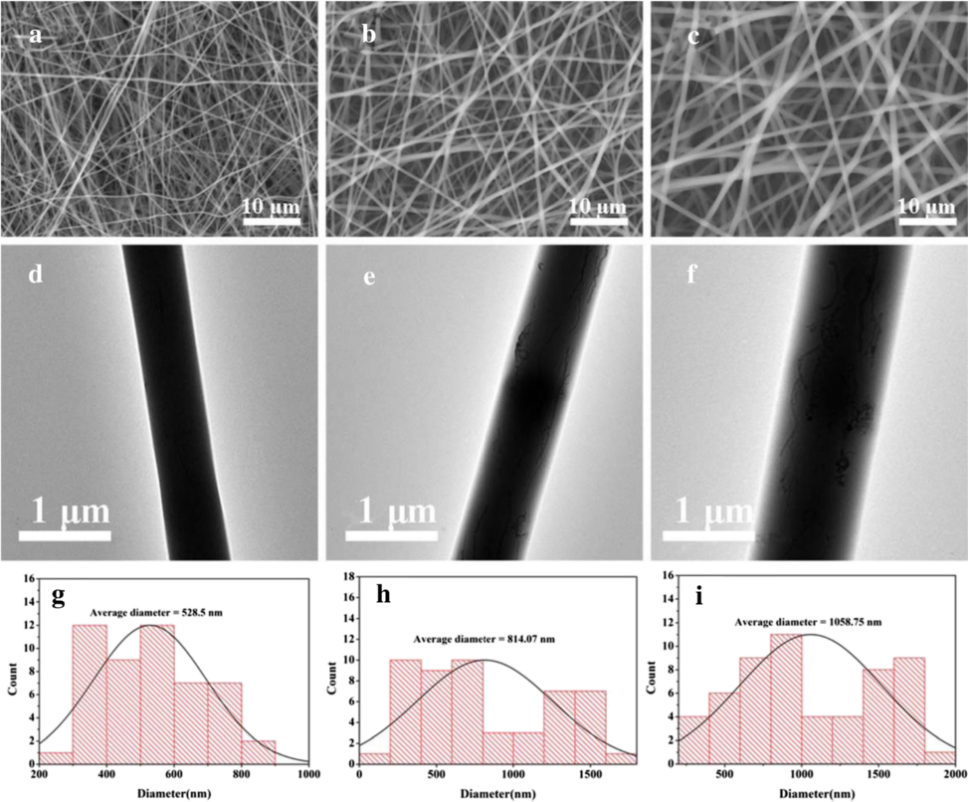
The morphology and diameter distributions of PLGA and PLGA/DOX@CNTs composite nanofibers. SEM images of **a** PLLA/1.5 % DOX@0.5 % CNTs, **b** PLGA/1.5 % DOX@1 % CNTs, and **c** PLGA/1.5 % DOX@2 % CNTs nanofibers. **d**–**f** The corresponding TEM images of **a**–**c. g**–**i** The corresponding diameter distributions of **a**–**c**

It is known that the morphology of electrospun nanofibers is influenced by various parameters, such as the concentration of polymers, applied voltage, and flow rate of solution. For the organic/inorganic composite nanofibers, the morphology and the diameter can be dramatically affected by the content of inorganic component in polymers [20, 26]. Therefore, it is encouraged to investigate the effects of the content of CNTs on the nanofibrous morphology and diameter. Figure 2 shows the morphology and diameter distribution of the electrospun nanofibers. As shown in Fig. 2a, it can be clearly seen that a smooth surface morphology and relatively uniform fibrous diameter were observed in PLGA/0.5 % CNTs, with the average diameter of 528.5 nm (Fig. 2g). When the content of CNTs was 1 % (Fig. 2b), a similar fiber morphology was seen. While the fiber diameter slightly increased, the average diameter of PLGA/1 % CNTs was 814.1 nm (Fig. 2h). When the content of CNTs was up to 2 %, the fibers of PLGA/2 % CNTs were changed to be swollen to a certain extent (Fig. 2c), but the fiber morphology still maintained. Accordingly, the average diameter of PLGA/2 % CNTs was increased to 1058.8 nm (Fig. 2i). As a result, the average diameter of the fibers gradually increased with increasing content of CNTs, which can be attributed to the increase in viscosity of the solution after the addition of a large amount of CNTs [26, 27]. Additionally, the TEM images of Fig. 2d–f clearly shows that CNTs can be homogenously distributed within the nanofibers, and more CNTs appeared with the increase of the CNTs content. Hence, 2 % of CNTs was an alternative in the following experiments.

**Fig. 3:**
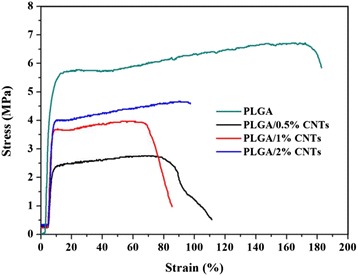
Typical tensile stress-strain curves of electrospun PLGA nanofibers, PLGA/0.5 % CNTs, PLGA/1 % CNTs, and PLGA/2 % CNTs composite nanofibers

Figure 3 shows the typical tensile strain-stress curves of neat PLGA and the PLGA/CNTs composite nanofibers with different CNTs contents. The mechanical properties of these nanofibers are summarized in Table 1. It can be found that the addition of CNTs affected the mechanical properties of the composite nanofibers, whose tensile strength, elongation at break, and Young’s modulus were smaller than that of the neat PLGA nanofibers. This tendency was similar with the mesoporous silica nanoparticles-embedded nanofibers, which may result from the aggregation of CNTs in hybrid nanofibers and poor interfacial adhesion between the CNTs and the PLGA matrix [22]. Furthermore, among the composite nanofibers, the tensile strength increased with the increasing of CNTs contents, which was basically in agreement with the data of the elongation at break. However, the Young’s modulus of PLGA/1 % CNTs was greater than that of the other composite nanofibers. For example, the Young’s modulus for composite nanofibers with 1 % of CNTs content was 145.53 MPa as compared to 76.50 MPa for PLGA/1 % CNTs and 142.25 MPa for PLGA/2 % CNTs nanofibers.

**Table 1:** Tensile mechanical properties of PLGA nanofibers and PLGA/CNTs nanofibers

CNTs content (%)	Tensile strength (MPa)	Elongation at break (%)	Young’s modulus (MPa)
0	6.78 ± 1.00	177.38 ± 24.82	170.71 ± 46.22
0.5	2.87 ± 0.30	65.43 ± 18.32	76.50 ± 2.30
1	4.08 ± 0.11	78.13 ± 12.94	145.53 ± 8.67
2	4.45 ± 0.40	97.33 ± 2.10	142.25 ± 3.30

**Fig. 4:**
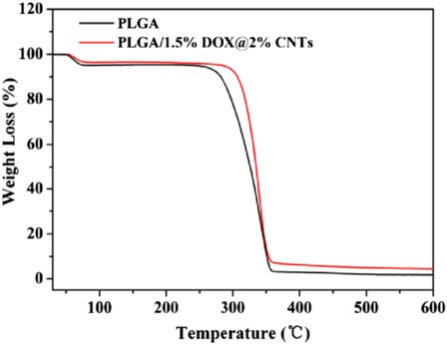
TGA thermograms of neat PLGA and PLGA/1.5 % DOX@2 % CNTs composite nanofibers

TGA was used to examine the thermal properties of obtained electrospun nanofibers and confirm the successful incorporation of drug-loaded CNTs within the composite nanofibers. As seen from Fig. 4, a moderate loss in weight happened within 100 °C, which was due to the vaporization of residual water in nanofibers. Then, a sharp weight loss between 250 and 400 °C can be clearly observed. The weight loss percentages of PLGA and PLGA/1.5 % DOX@2 % CNTs were calculated to be 2.5 and 4.1 %, respectively, suggesting that about 2 % of CNTs had been successfully incorporated into the fibers, which was almost equal to the initially added weight of CNTs. Additionally, the TGA results also revealed the effect of added CNTs on the thermal stability of composite nanofibers. The onset degradation temperature (*T*_onset_) of the PLGA nanofibers was 247 °C, whereas the *T*_onset_ value (270 °C) of PLGA/1.5 % DOX@2% CNTs was prominently increased, indicating that the addition of CNTs can improve the thermal stability of the composites, which was similar to the results obtained by Qiu et al. [22].

### DOX Loading and Release

**Fig. 5:**
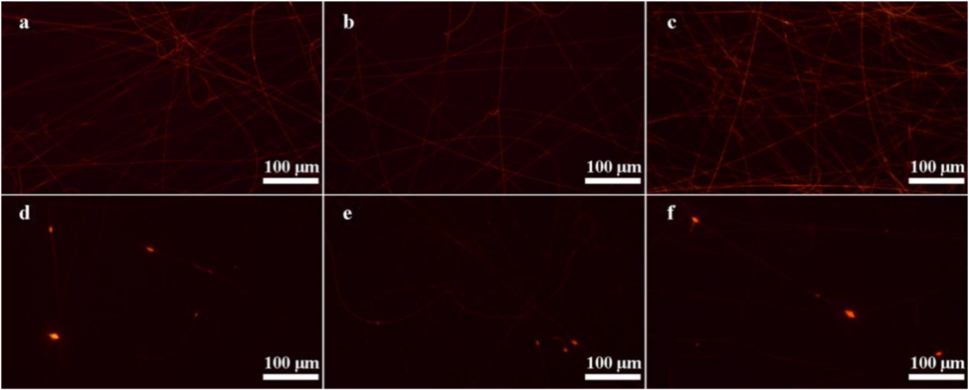
Fluorescent images of PLGA/DOX and PLGA/DOX@CNTs composite nanofibers. **a** PLGA/0.5 % DOX, **b** PLGA/1.0 % DOX, **c** PLGA/1.5 % DOX, **d** PLGA/1.5 % DOX@0.5 % CNTs, **e** PLGA/1.5 % DOX@1 % CNTs and **f** PLGA/1.5 % DOX@2 % CNTs composite fibers

To investigate the DOX loading and releasing behavior of composite nanofibers, DOX-loaded CNTs were firstly prepared. The high loading percentage (81.5 %) was attributed to the special sp^2^-hybridized carbon surfaces and a large surface area of CNTs [28]. Then, the DOX-loaded CNTs were incorporated into PLGA nanofibers via electrospinning. Fluorescence microscopy was used to observe the DOX-loaded composite nanofibers via the red fluorescence of DOX (Fig. 5). From Fig. 5a–c, we can see that all the PLGA/DOX samples displayed a uniform red fluorescence, indicating the homogeneous dispersion of DOX in the nanofibers. Similarly, the red fluorescence of DOX can be clearly observed in the PLGA/DOX@CNTs nanofibers. Nevertheless, some local red spots appeared as compared to their PLGA/DOX counterparts (Fig. 5d–f), which may be caused by the partial aggregation of CNTs in the nanofibers. This result also revealed that the DOX@CNTs can be incorporated into PLGA nanofibers with relatively uniform distribution within the nanofibers.

**Fig. 6:**
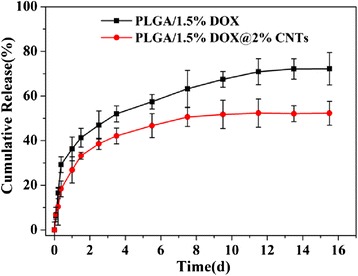
The cumulative DOX release profiles from PLGA/1.5 % DOX and PLGA/1.5 % DOX@2 % CNTs nanofibrous mats

The DOX release behavior was investigated under stimulated physiological environment (pH = 7.4) at 37 °C. For comparison, the release profiles of PLGA/1.5 % DOX and PLGA/1.5 % DOX@2 % CNTs electrospun nanofibers containing the equivalent DOX contents were studied. As shown in Fig. 6, both electrospun mats exhibited relatively rapid release rate at the beginning 12 h, which was resulted from the fast diffusion of DOX molecules closed to the nanofibers surface. Afterwards, the DOX was released more slowly from CNTs-incorporated mats than that from the neat PLGA nanofibers, indicating the CNTs could effectively protect drug molecules from premature leakage, which was beneficial to decrease side effect of anticancer drugs. Moreover, the release behavior of PLGA/1.5 % DOX@2 % CNTs sustained for 8 days with nearly 50 % of total drug released. The steady and tardy DOX release endows the hybrid mats with a desired therapeutic concentration of anticancer drug over an extended period of time, which was efficient to kill cancer cells.

### In Vitro Antitumor Effect

**Fig. 7:**
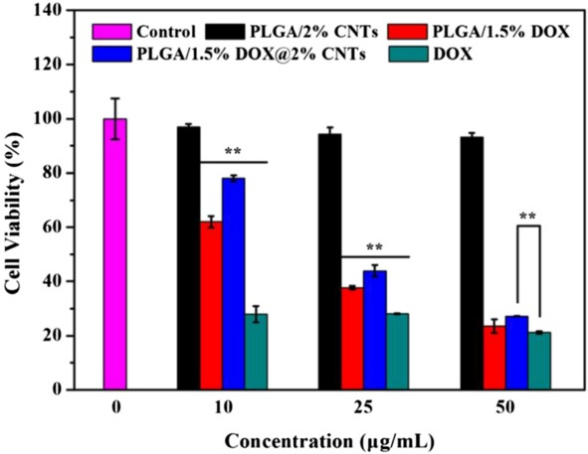
Cell viability of HeLa cells treated with different samples with DOX concentrations ranging from 10 to 50 μg/mL

In antitumor application of drug-containing materials, a high antitumor effect was needed. Therefore, we next investigated the antitumor activity of the DOX upon release from DOX-loaded composite nanofibers by MTT assay. Figure 7 shows the cell viability of HeLa cells treated with different samples with DOX concentrations ranging from 10 to 50 μg/mL for 24-h incubation. As compared with the control (medium only), it was obvious that the neat PLGA/CNTs nanofibers did not show any cytotoxicity to HeLa cells within the tested concentrations. But for other groups (PLGA/1.5 % DOX, PLGA/1.5 % DOX@2 % CNTs and free DOX), a dose-dependent cytotoxicity was observed. For free DOX group, it showed a statistically significant higher inhibition effect than other groups at all the measured concentrations (*P* < 0.01). However, PLGA/1.5 % DOX@2 % CNTs composite nanofibers showed a statistically significant lower inhibition effect than PLGA/1.5 % DOX and free DOX when the DOX concentration was less than 50 μg/mL (*P* < 0.01), which was due to the slow release rate of DOX from the composite nanofibers. Additionally, PLGA/1.5 % DOX and PLGA/1.5 % DOX@2 % CNTs composite nanofibers started to have apparent cytotoxicity only at DOX concentration of 25 μg/mL or above. It is noted that the inhibition effect of PLGA/1.5 % DOX@2 % CNTs composite nanofibers was equal to PLGA/1.5 % DOX when the DOX concentration was 50 μg/mL but lower than the free DOX. Although PLGA/1.5 % DOX@2 % CNTs composite nanofibers have not shown the relatively prominent anticancer efficacy compared with the PLGA/1.5 % DOX and free DOX group from the MTT assay, but we expected that a long-term growth inhibition in cancer cells could be achieved with the sustained DOX release from this matrix.

**Fig. 8:**
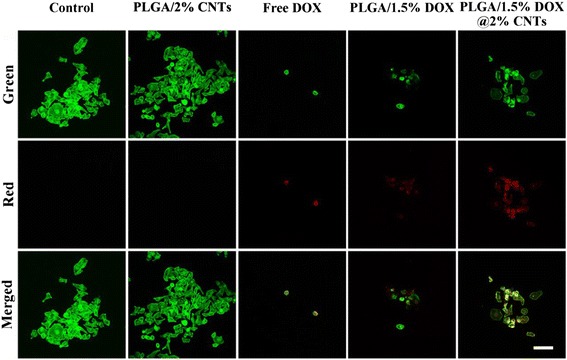
Confocal laser scanning microscopy images of HeLa cells treated with PLLA/2 % CNTs, free DOX, PLLA/1.5 % DOX, and PLLA/1.5 % DOX@2 % CNTs. DOX concentration was 25 μg/mL. The *red fluorescence* indicates the released DOX. The *green fluorescence* represents Alexa Fluor 488® phalloidin-stained F-actin. *Scale bars* = 100 μm

CLSM imaging technology was further employed to gain more insights into the therapeutic effect caused by PLGA/DOX@CNTs. The Alexa Fluor® 488 phalloidin was used to label cytoskeleton and the red fluorescent nature of DOX facilitated the in situ observation. As shown in Fig. 8, it is worth noting that the free DOX, PLGA/1.5 % DOX and PLGA/1.5 % DOX@2 % CNTs could effectively inhibited the growth of HeLa cells, because the drug treated cells displayed apparent morphological features of apoptosis after treatment for 24-h compared with control. The cells incubated with PLGA/2 % CNTs nanofibers showed no significant abnormality on the morphology, indicating that the cytotoxicity was associated with the DOX molecule, not the nanocarrier.

## Conclusions

In summary, we have reported a very effective and reproducible route to prepare a drug delivery system based on PLGA/CNTs composite nanofibers. The highly uniform and smooth nanofiber is successfully designed and developed. By combining the CNTs, as a proof-of-concept, we demonstrated that the PLGA/CNTs composite nanofibers could be used for the sustained release of the anticancer drug molecule DOX, which is important for biomedical applications requiring the drug molecule to maintain long-term anticancer efficacy. And the release profiles clearly indicated that the DOX can be loaded in the inner cavities or on the outside surface of the CNTs weakening the initial burst release of DOX. In addition, our results indicated that the prepared PLGA/DOX@CNTs nanofibers platform could effectively inhibit the cell viability of HeLa cells in vitro. Our results shed light on a promising use of the PLGA/DOX@CNTs composite nanofibers as a long-term drug release nanoplatform for chemotherapy in clinical cancer treatment.
